# Molecular Epidemiology and Clinical Characteristics of Hepatitis B Identified through the French Mandatory Notification System

**DOI:** 10.1371/journal.pone.0075267

**Published:** 2013-09-25

**Authors:** Vincent Thibault, Syria Laperche, Valérie Thiers, Sophie Sayon, Marie-José Letort, Elisabeth Delarocque-Astagneau, Denise Antona

**Affiliations:** 1 Virology Laboratory, Hôpital Pitié-Salpêtrière, Assistance Publique (APHP), and Pierre et Marie Curie University, Paris, France; 2 National reference center for hepatitis B and C in blood transfusion, National Institute of blood transfusion, Paris, France; 3 Virology Department, Institute Pasteur, Paris, France; 4 Infectious Diseases Department, National Institute for Public Health Surveillance (Institut de veille sanitaire), Saint-Maurice, France; 5 Emerging Diseases Epidemiology Unit and Pharmacoepidemiology and Infectious Diseases Unit, Institut Pasteur, Paris, France; The University of Hong Kong, Hong Kong

## Abstract

**Background & Aims:**

Strains responsible for acute hepatitis B infections (AHB) in France have not been characterized. This study was first designed to analyze the molecular epidemiology of AHB and second to describe the differences between AHB and chronic hepatitis B (CHB) exacerbations.

**Methods:**

This prospective study was based on the French mandatory notification system for AHB. 147 samples corresponding to declared cases were shipped to a central laboratory for classification as AHB or CHB according to the level of anti-HBc IgM and anti-HBc avidity.

**Results:**

Based on biological marker values and file examination, 75 cases (59%) were classified as AHB. Independently of the acute or chronic status, genotype A (57%), D (22%) and E (14%) were the most prevalent and no phylogenetic clustering was observed among HBV sequences (n=68). Precore or basal core-promoter variants were not particularly associated with disease severity but were more prevalent in CHB. No antiviral resistant strains or immune-escape HBsAg was observed. HBV viral loads in AHB or CHB were comparable but with opposite distributions. ALT levels reached 10 times the upper normal value in 94% of AHB but only in 24% of CHB.

**Conclusions:**

After rigorous classification, no major difference at the genetic level was found between HBV strains isolated from AHB and CHB. Absence of potentially deleterious variant detection is reassuring. When based upon HBsAg and anti-HBc IgM determination, AHB notification may falsely include more than 40% CHB, leading to an important risk of bias in national surveillance programs of AHB.

## Introduction

It is estimated that about 360 million individuals worldwide are chronically infected by HBV and at risk of serious hepatitis B related complications [1]. The yearly death toll attributed to HBV-related diseases reaches approximately 620 000 individuals [2]. Fortunately, 92% of all countries have implemented vaccination during childhood with an overall coverage of 69%. In France, considered a low HBV prevalence country with an HBsAg prevalence of 0.65% [95% CI: 0.45-0.93], vaccination has suffered bad press for the past 15 years and immunization coverage in 1 year old infants was still below 50% in 2007 [3,4].

Molecular epidemiology of HBV infections in France have mostly been studied in the context of CHB outpatient clinics but no information is available for incident cases [5,6,7]. Precise molecular characterization is necessary to identify HBV antiviral resistant strains or immune escape variants that could spread within a vaccinated population [8].

In France, mandatory notification of acute cases of HBV infection (AHB) has been implemented since 2003 and, from around 158 notifications of incident cases each year, it has been calculated that approximately 2600 HBV infections could be contracted in France per year [9]. An acute case has to be notified when anti-HBc IgM are detected for the first time in a patient, or, if anti-HBc IgM were not tested, when HBsAg was detected in the context of an acute hepatitis defined by clinical symptoms and important increase of transaminases.

Identification of AHB, by opposition to chronic hepatitis B (CHB) exacerbation, is often very tricky due to the lack of robust and specific acute phase markers [10]. Among available markers, only high level of anti-HBc IgM and low anti-HBc IgG avidity are often found during acute course of HB, yet their use is not well standardized and they are seldom proposed as routine assays [11,12,13,14]. Consequently, a precise definition for acute case notification is, at most, a second best choice.

A one year study based on the French mandatory notification system was conducted with the main purpose to characterize at the molecular level the strains responsible for acute infections, to identify possible clinically relevant variants and to analyze the clinical and biological factors associated with the disease course. In order to stringently dissociate AHB from CHB, a strict algorithm based on anti-HBc IgM and avidity index was used [15,16].

## Methods

### Study organization

**Figure 1:**
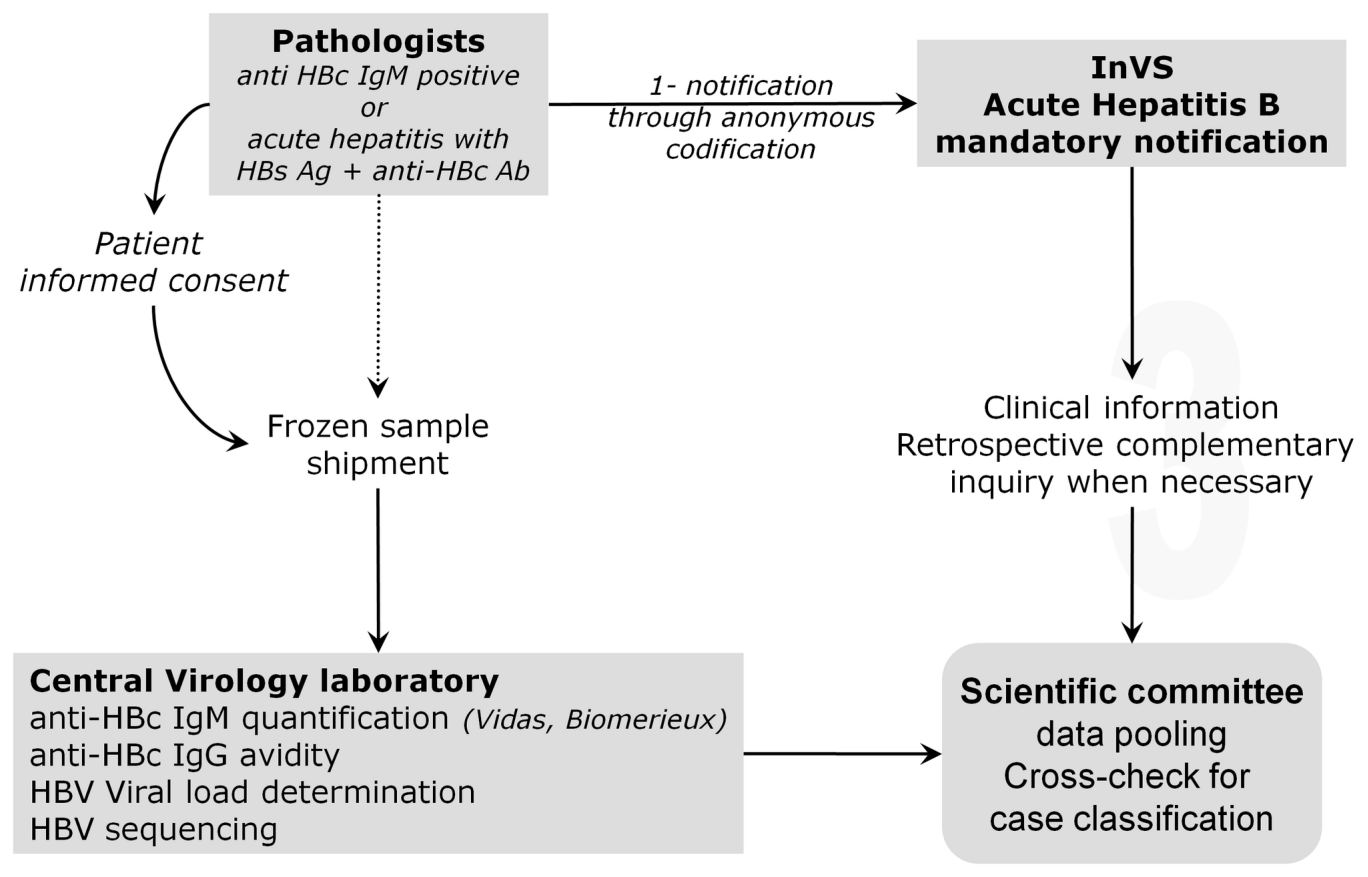
General organization of the French AHB registry.

**Figure 2:**
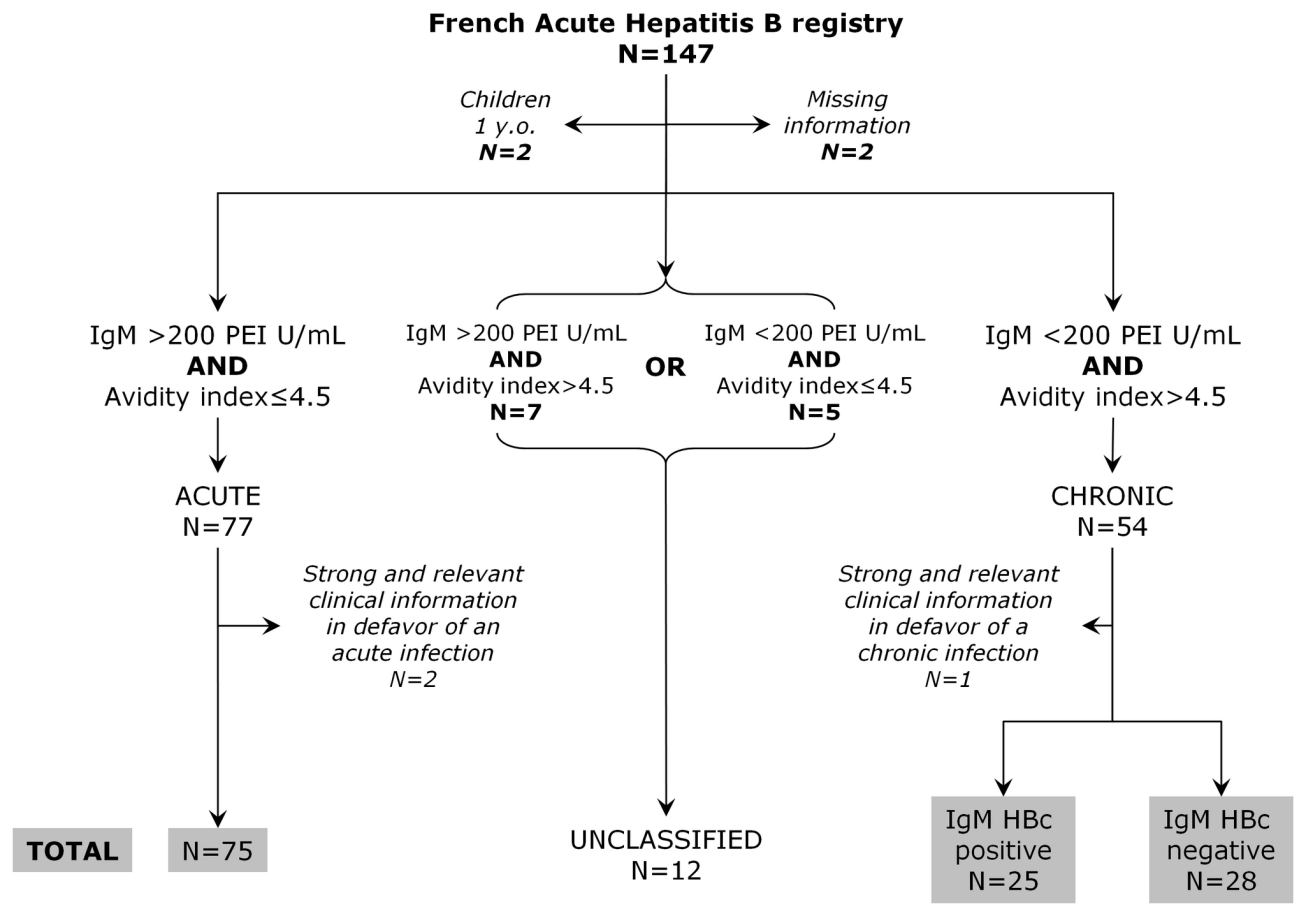
General flow chart of the study. Samples corresponding to notified AHB were classified according to both the values of HBc IgM and avidity index. Each biologically classified coherent case was thoroughly assessed for discrepancies with epidemiological and clinical information. When relevant information was not available physicians were interviewed on an individual basis.

As depicted on Figure 1, this prospective study was based on the French mandatory notification requiring that any acute infection identified by a pathologist should be anonymously notified to "Institut de Veille Sanitaire" (InVS). Clinical and epidemiological information (including exposures in the 6 months prior to onset of symptoms and risk factors: occupational, intravenous drug use, nosocomial, other parenteral risks, sexual, in-house, perinatal and others) are further completed by the physician in charge. From February 2007 to April 2008, all French pathologists were called upon to participate. They were asked to obtain informed consent of any newly diagnosed patient, and upon patient’s agreement to ship at least 1mL of frozen serum to the central laboratory in Paris. As recommended by the French health authorities for this type of study, all pathologists gave verbal information to the patients about their participation to the study and the possibility to be removed from it at any time. Once they obtained each patient’s consent, pathologists had to fill a specific registration form using an anonymous code per patient and to ship it with the serum sample. This study conforms to the ethical guidelines of the 1975 Declaration of Helsinki and its design was approved by the ethics committees "Commission nationale informatique et libertés" (CNIL) and "Comité Consultatif National d’Ethique" (CCNE). Each AHB notification was examined for correctness and missing information was collected by InVS through telephone calls. Samples were received for all the notifications received during the study period. Following pooling of all data, the scientific committee composed by all authors of this publication agreed upon final classification of each case based on IgM level, avidity index and epidemiological records. Any case that did not match all criteria was excluded as outlined on Figure 2.

### Virological analyses

Anti-HBc IgM quantification was performed using Vidas HBc IgM-II assay from bioMérieux (Marcilly-l’Etoile, France) following manufacturer’s recommendations. Results were expressed as PEI IU/mL; values below or equal to 5 PEI IU/mL were considered negative.

Anti-HBc avidity was determined as previously described [16]. Briefly, Anti-HBc measurement was performed using Monolisa anti-HBc-Plus (BIORAD, Marnes-La-Coquette, France). A dissociation index was calculated using the ratio of the readings obtained with/without 6M urea as dissociating agent. The relative amount of anti-HBc antibody was assessed using the ratio between the signals obtained for each sample over the assay cut-off and a composite avidity index was calculated taking into account both the dissociation index and the relative anti-HBc level. An avidity index below or equal to 4.5 was identified as predictive for recent hepatitis B, typically within the previous 3 months.

HBV viral load was determined using Abbott HBV RealTime assay (Abbott Molecular, Rungis, France) with a quantification range from 10 to 1 billion IU/mL.

For all samples, full length genome amplification was attempted using Günther’s method [17]. If complete genome amplification failed, partial genome amplification was attempted using several primer pairs distributed throughout HBV genome. Amplified fragments were sequenced using Bigdye Terminator V3.0 (ABI, Les Ulis, France) and genotypes were determined by phylogenetic analysis using reference Genbank sequences [18].

Resistance conferring polymerase substitutions, HBsAg amino-acid changes and precore/basal core promoter mutations were individualized for each generated sequence.

### Case classification

To be retrospectively classified as acute, a case should have a positive HBsAg detected for the first time in the reporting laboratory (basis for notification) and both an anti-HBc IgM value over 200 PEI U/mL, as measured in the central laboratory and an avidity index below or equal to 4.5. Cases not fulfilling these criteria were considered chronic.

The anti-HBc IgM limit of 200 PEI IU/mL was chosen arbitrarily as it corresponds to the upper range of quantification of the assay. According to the most comprehensive study on this issue, the positive predictive value for AHB using this cut-off is around 91% [12]. The avidity index marker was validated on commercial and in-house HBV seroconversion panels (31 samples) and a value of 4.5 was only observed in samples that were detected HBsAg-positive within the previous three months. By combining both markers, it was admitted that the chances to biologically misclassify a sample was minimal.

### Statistical analysis

Statistical analyses were performed by chi-square and Fisher’s exact tests for categorical variables and the Mann-Whitney U-test or Student’s t-test, and ANOVA for quantitative variables. P values below 0.05 were considered significant. Analyses were performed using StatView 5.0 software (SAS, Cary, NC, USA) and Analyse-it for Microsoft Excel (version 2.20) (Analyse-it Software, Ltd. http://www.analyse-it.com/).

## Results

### Stringent classification of acute and chronic hepatitis B

Figure 2 summarizes the classification process applied to the 147 samples received for viral characterization. Four notifications were not considered; 2 because they concerned young children and the biological assays were only validated for adults and 2 because of missing relevant Information. Twelve samples with discrepant results between anti-HBc IgM and avidity index were not considered for further analysis. Remaining files were reviewed and led to identification of 128 cases (75 AHB, 53 CHB). Anti-HBc IgM was not detected in 53% (28) of CHB.

### Case main characteristics

Overall, the sex ratio (M/F) was 1.5 and comparable across all groups. However, age and sex distributions were different in acute and chronic cases, with AHB predominantly affecting younger women (median age=33) and men over 40 (median age=42), p=0.0175. AHB presented with higher ALT level than CHB (p<0.001) reaching a median value of 2254 IU/L (IQR: 1381-3539) versus 70 (IQR: 28-451) and with more often jaundice (57.6% vs. 7.6%; p<0.001), independently of gender. In AHB, ALT elevation distribution was different according to age (p<0.001) with the highest values observed between 20 and 40 years of age (median: 2670 IU/L). Although only 6 individuals were concerned, ALT elevation was not remarkably high in patients over 60 years (median: 93 IU/L).

The main at risk exposures were collected but there were too few cases in each group to draw firm conclusions. Among identified risk factors, the two most prevalent were sex (20.2%) and men having sex with men (13.8%) with an equal distribution in AHB and CHB.

### Virological study

**Figure 3:**
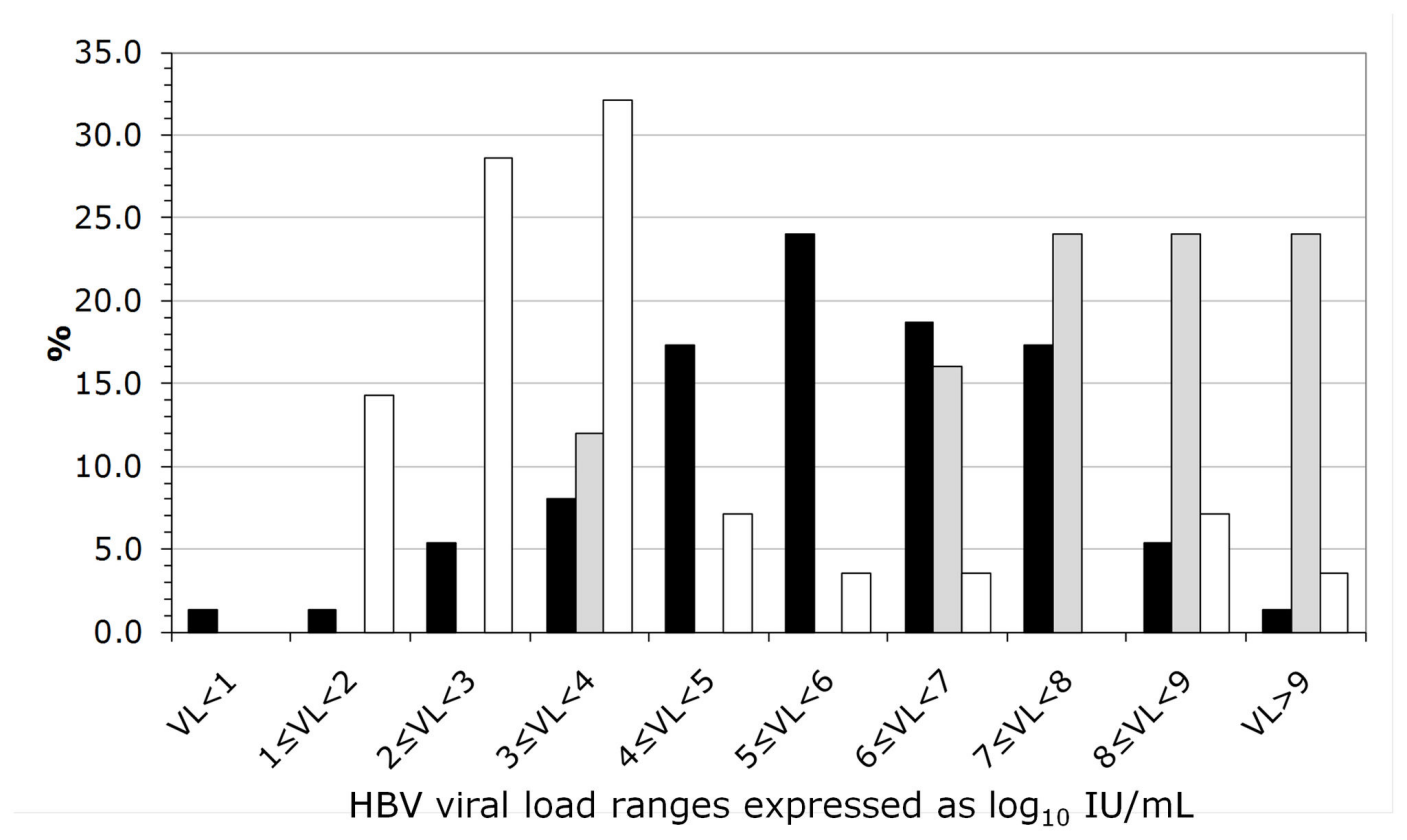
Distribution of HBV-VL (log_10_ IU/mL) according to disease course. Black bars represent acute cases and gray bars anti-HBc IgM positive chronic cases and open bars anti-HBc IgM negative chronic cases.

Even though median viral loads (HBV-VL) were not significantly different between acute (5.7 log IU/mL, IQR: 2.23) and chronic cases (med. 5.6 log IU/mL, IQR: 5.05), the distribution was highly different (Figure 3). HBV viral load was significantly different (p<0.0001) between the 28 CHB without anti-HBc IgM and the 25 CHB with anti-HBc IgM, with values of 3.1 (1.28-9) and 7.9 (3.34-9.99) log IU/mL, respectively. No association was found between ALT elevation and HBV-VL in AHB while high ALT were only observed in chronic carriers with HBV-VL over 6 log_10_ IU/mL. A correlation (p=0.028, r^2^=0.131) between ALT and VL was observed in chronic cases.

The most frequently detected genotypes were A (44.5%), D (21.9%) and E (14.1%) while genotypes F, C, B and G represented less than 20% of the sequenced samples. Genotype distribution was not different between acute and chronic cases (Table 1) or according to risk factors. In AHB, there was a trend (p=0.05) for different ALT elevation distribution according to genotype. ALT increase associated with genotype D-AHB (med. 1429 UI/L) was slightly lower than for other genotypes (p=0.017).

**Table 1:** Main patients’ characteristics.

	**All**	**Acute**	**Chronic**	**Chronic**	p
			**HBc IgM positive**	**HBc IgM negative**	
n	**128**	**75**	**25**	**28**	
*Age (range)	39.5 (13-84)	40 (16-82)	46.8 (13-84)	36 (17-71)	0.04
Male gender (%)	83 (64.8)	50 (66.7)	19 (76)	14 (50)	
**Liver disease markers**					
ALT (range) IU/L	1492 (4-9625)	2321 (35-9625)	132 (9-1467)	38 (4-2209)	<0.001
Icterus	65.2% (60/92)	57.6% (53/70)	36% (5/14)	25% (2/8)	<0.001
ALT distribution % (n)					<0.001
≤35 UI/L	12 (13)	1.4 (1)	19 (4)	50 (8)	
35<ALT≤350	17.6 (19)	4.2 (3)	47.6 (10)	37.5 (6)	
350<ALT≤1750	26.9 (29)	29.6 (21)	33.3 (7)	6.2 (1)	
ALT>1750	43.5 (47)	64.8 (46)	0 (0)	6.2 (1)	
**Virological markers**					
anti-HBc IgM (PEIU/mL)	>200 (0-200)	>200	44 (8-164)	0 (0-4)	<0.001
Avidity index (range)	3.2 (0.2-8.9)	1.7 (0.2-4)	6.9 (4.9-8.4)	7.3 (5.3-8.9)	<0.001
HBV-VL (range)	5.6 (0.8-10)	5.7 (0.8-10)	7.9 (3.3-10)	3.1 (1.3-9)	
VL <2000 IU/mL % (n)	21.1 (27)	10.7 (8)	35.8 (19)	35.8 (19)	<0.001
**Variant distribution % (n**)					
Genotype A	48.3 (57)	50 (37)	36 (9)	39.3 (11)	
Genotype B	1.7 (2)	1.3 (1)	4 (1)	0 (0)	
Genotype C	5.1 (6)	4 (3)	8 (2)	3.6 (1)	
Genotype D	23.7 (28)	20.3 (15)	20 (5)	28.6 (8)	
Genotype E	15.3 (18)	17.6 (13)	8 (2)	10.7 (3)	
Genotype F	5.1 (6)	6.8 (5)	4 (1)	0 (0)	
Genotype G	0.8 (1)	0 (0)	0 (1)	3.6 (1)	
A1762T BCP	23 (20)	14.8 (9)	28 (7)	14.3 (4)	0.01
G1764A BCP	26.4 (23)	19.7 (12)	28 (7)	14.3 (4)	0.04
G1896A PC	14.6 (15)	8.7 (6)	20 (5)	14.3 (4)	0.02

* Unless otherwise stated, median values are indicated for quantitative variables.

Overall, basal core promoter (BCP) mutation A1762T or G1764A were detected in 20% and 23% of the cases, respectively, while pre-core (PC) PC-G1896A was present in only 15% of tested samples (Table 2). PC, but not BCP, mutation prevalence was linked to genotype (p<0.0001). PC mutations were never detected in HBV-A but in 22.2% HBV-E and 33.3% HBV-D. Distribution of PC and BCP mutations were significantly linked to disease course. For instance, PC-G1896A and BCP variants were found 3 (p=0.017) and 2.8 (p=0.012 for A1762T) times more often in chronic carriers than in AHB, respectively. CHB patients, but not AHB, carrying PC G-to-A variants were older (med. 45y.) than those with wild-type virus (med. 33y.; p=0.047). When taking ALT rise as marker of liver disease severity in acute or chronic cases, no difference was observed according to the presence of 1896 or 1762/1764 variant.

**Table 2:** Distribution of BCP/PC mutations according to genotype.

Mutation	**A1762T**		**G1764A**		**G1896A**	
Total	23% (20/87)		26.4% (23/87)		14.6% (15/103)	
HBV Status	**Acute**	**Chronic**	**Acute**	**Chronic**	**Acute**	**Chronic**
n	**61**	**26**	**61**	**26**	**69**	**34**
Genotype						
A	16.7 (5)	44.4 (4)	20 (6)	33.3 (3)	0 (0)	0 (0)
B	0 (0)	0 (0)	0 (0)	0 (0)	100 (1)	100 (1)
C	33.3 (1)	66.7 (2)	33.3 (1)	66.7 (2)	0 (0)	33.3 (1)
D	7.7 (1)	12.5 (2)	15.4 (2)	25 (2)	14.3 (2)	60 (6)
E	20 (2)	60 (3)	20 (2)	60 (3)	23.1 (3)	20 (1)
F	0 (0)	100 (1)	25 (1)	100 (1)	0 (0)	0 (0)

Very few samples presented amino-acid substitution known to modify HBsAg immunogenicity and no difference could clearly be identified between CHB and AHB. No residue change associated with treatment resistance (rt169, 173, 180, 181, 184, 202, 204, 215, 236 and 250 positions) was observed in the entire cohort.

**Figure 4:**
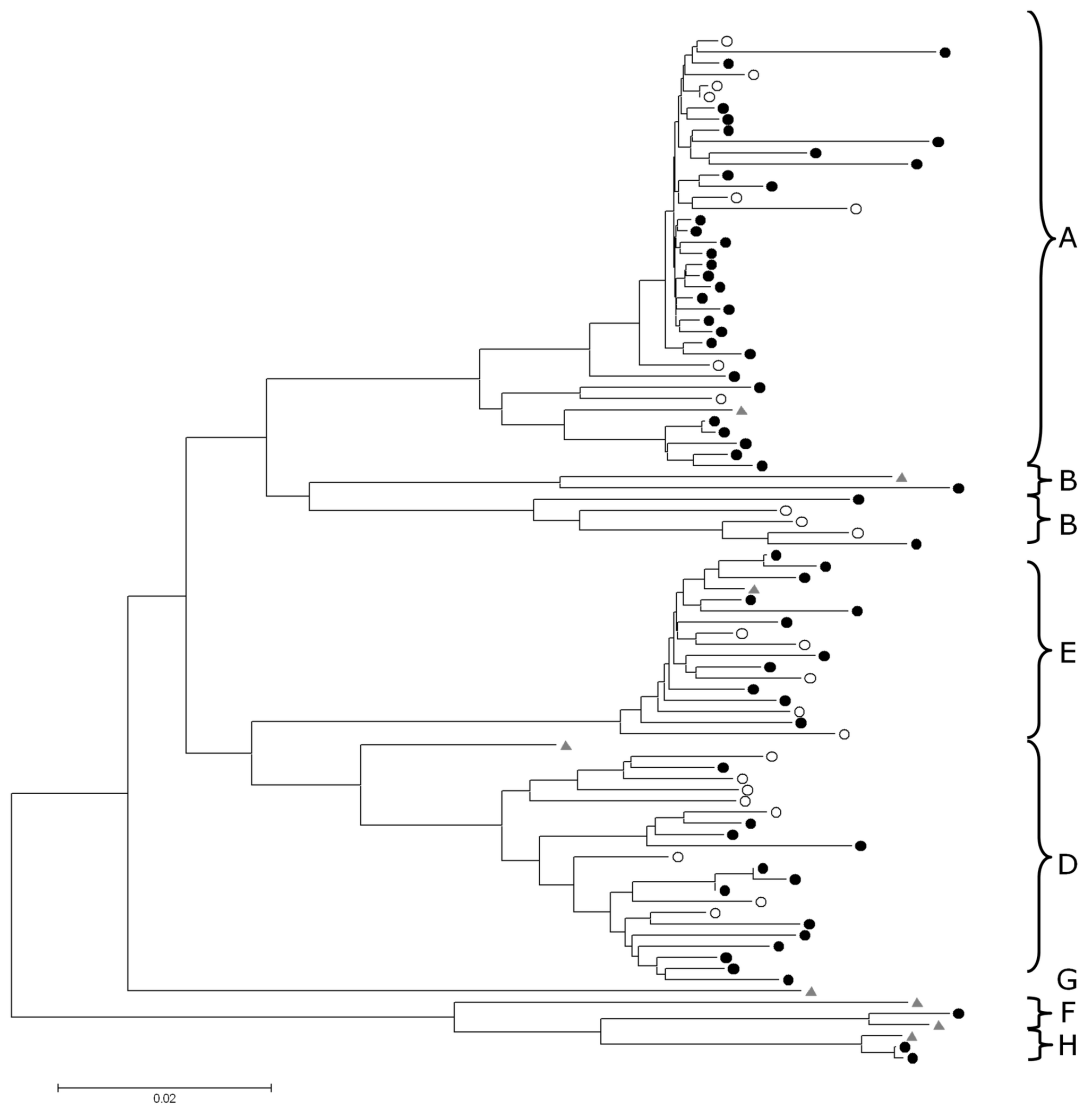
Phylogenetic tree obtained from 92 full length sequences. The evolutionary history was inferred using the Neighbor-Joining method with a bootstrap test (500 replicates); bootstrap values are shown next to the branches. Sequences from 60 acute cases (black dots) and 24 chronic cases (open dot) and 8 reference sequences (grey triangles) were analyzed.

A phylogenetic tree was constructed using the 84 sequences (60 AHB, 24 CHB) covering more than 90% of HBV genome length together with Genbank reference sequences. Distribution of acute and chronic cases was harmonious along the entire tree and within each genotype group (Figure 4). Analysis of genetic distances between groups using the Jukes-Cantor model confirmed the tree distribution; diversity within each genotype between AHB and CHB (range 0.0197-0.039) did not differ from the overall diversity within the same genotype group (0.0225-0.0362).

## Discussion

This prospective study based on the French mandatory notification system for AHB represents probably the most exhaustive approach to obtain virological information on acute cases and to gather unbiased virological information on patients who were not previously screened and therefore not treated for HB. Yet, it should be taken into consideration that notifications are under reported by physicians (level of reporting estimated at 23% of actual acute cases [9]) and that most of AHB are asymptomatic leading to underestimating the disease burden. Additionally, correct classification of AHB has always been a difficult task. Indeed, depending on each national notification system, the criteria to notify an AHB can be notably different and could lead to 40% misclassification of CHB as AHB [19]. Because perfection seems very difficult to reach for correct classification of acute cases, several strategies have been developed based on clinical information that sometimes achieves reasonable positive predictive values [20]. In order to avoid as much as possible any clinical bias, our strategy was solely based on biological markers. Thus, the first screen was from each notifying laboratory and the second screening process was performed by a central laboratory testing both anti-HBc IgM and anti-HBc avidity. Although anti-HBc IgM is not highly predictive of AHB, several studies have shown that high values are almost exclusively encountered during the early phase of infection and are seldom seen during reactivation [11,12,13,14]. Unfortunately, lack of standardization of commercialized assays and absence of any valid clinical threshold make use of this marker quite unreliable [12]. The choice was made to use a single assay in a unique central laboratory and to classify samples with values above 200 PEI U/mL as from AHB [11,13,14]. In parallel, anti-HBc avidity was determined. We have previously shown that a value below 4.5 of a composite index based on anti-HBc antibody relative concentration and avidity was highly predictive of the recent apparition of HBsAg within the previous three months [16]. By simply measuring anti-HBc avidity, Rodella et al. reached also similar conclusions [15]. Using this biological screen (Figure 2), more than 90% of the notifications could be classified, 8.6% (n=12) having discordant profiles between anti-HBc IgM and avidity index. Among the remaining 131 cases, 59% (n=77) had a homogenous biological profile for acute infection but 2 (2.6%) were excluded because clinical information was firmly in disfavor of an acute episode. These two discordances clearly illustrate the limit of a classification solely based on biological markers but are in agreement with the observed specificity and sensitivity of these markers [12,15,16]. By choosing such stringent criteria, we feel confident that our classification is, as accurately as possible, representative of the true natural history. Noteworthy, the gold standard for classification could have been to perform a longitudinal study for all patients, looking at the loss of HBsAg within the following 6 months, an evolution almost exclusively observed after acute infection. Unfortunately, our study was based on the mandatory notification that does not include any follow-up visit and it was not possible to efficiently collect such information. Future studies on this topic should certainly consider documenting the infection natural evolution within the next 6 months in order to retrospectively classify accurately notified cases.

According to three independent studies in French CHB patients, genotypes A, D and E, classified as decreasing prevalence, are the most prevalent and, generally, typical Asian genotypes B and C account for 5.7 to 18.3% [5,6,7]. Low incidence of these last genotypes coupled with a high incidence of HBV-A were also observed in Holland and in England and Japan acute cases [21,22,23]. Accordingly, a recent study looking at HBV from asymptomatic blood donors indicated a high prevalence of genotype D (43%) but highlighted a high number of genotype A in recently acquired HBV [24]. Despite the fact that no association between genotype and risk factor was observed in this study, our findings should help to promote prevention strategies after identification of risk factors associated with genotype A transmission, particularly in France where vaccination suffers bad press. The absence of difference in genotype distribution in AHB and CHB may indicate some kind of stability in HBV epidemiology in France between recent and older infections.

Particular variants, especially immune escape variants or treatment resistant strains were not identified in all investigated cases. While this finding can be interpreted as reassuring with regard to vaccine or current treatment efficacy, it could also mean a bias due to lack of detection of such specific variants by currently used HBsAg detection assays [25]. Yet, most assays currently used in France have been shown to correctly detect most naturally occurring variants. According to our experience, many detected HBsAg with our screening assay carry mutations known to affect HBsAg immunogenicity [26]. The hypothesis of an absence of detection of major variants seems therefore unlikely. In line with these findings and contrary to some studies, no resistant strains were detected in our population [27,28,29,30].

Although the main purpose of the study was not to study the clinical severity of the disease at the time of diagnosis, information on ALT elevation and presence of an icterus was available for the majority of patients. Most patients were seen in a context of symptomatic disease: icterus in 65.2% of them and median ALT value of 1492 IU/L. Interestingly, cases classified as acute had significantly higher ALT values and were more frequently jaundiced as compared to CHB. Overall, HBV-VL was not predictive of disease severity and the highest viral loads were mostly observed in chronic carriers with ALT elevation, while acute course was characterized by viral loads ranging from 4 to 8 log_10_ IU/mL. Interestingly, and both in AHB or CHB, no difference in ALT elevation or presence of an icterus was observed according to the presence of BCP or PC mutation. These findings suggest that presence of PC or BCP mutations does not significantly influence acute disease severity. Indeed, data from the literature on this topic are usually controversial with many conflicting reports on the link between presence of these mutations and more severe evolution [31,32,33,34]. Contrary to most Asian studies, France is characterized by a large diversity of genotypes and the absence of association between disease severity and presence of PC/BCP mutation may be related to the relative genotypic diversity classically observed in our population. Yet, an absence of link between these mutations and disease severity was also reported in two studies from Taiwan and the USA [35,36]. Literature heterogeneity on clinical effect of these variants seems to indicate that multiple factors from the host and the virus are involved in the severity of the disease [37]. In agreement with our classification and current concepts on natural history of hepatitis B, PC/BCP mutations were found 2 to 3 times more often in chronically than in acutely infected patients [38,39]. An older age was also associated with presence of PC variants in CHB.

In summary, using stringent criteria to differentiate AHB from CHB flare, we were able to better identify the characteristics of strains responsible for new infections in France. Obviously, our study is biased as most, if not all, cases were tested for HBsAg in the context of a calling symptom while many asymptomatic acute infections remain undetected [9]. Yet, no other practical strategy was found to better address our objectives. Notification of acute cases based on classical markers may lead to an overestimation of new infections, with potential bias in incidence estimates. Thus, any notification program on acute hepatitis B should take into account the possibility to misclassify around one third of the symptomatic cases if stringent biological and epidemiological screens are not strictly applied.
